
JOURNAL INFORMATION
==============================
NLM Title Abbreviation: ScientificWorldJournal
Journal ID: ScientificWorldJournal
NLM Journal ID: 101131163
Journal ID: TSWJ

The Scientific World Journal

ISSN: 2356-6140
EISSN: 1537-744X
Publisher: Wiley

ARTICLE INFORMATION
==============================
PMCID: PMC6084404
PMID: 30147526
DOI: 10.1100/tsw.2001.226
Article ID: 564235
Article version: 1
Subjects: Short Report

Rapid Onset of Nucleolar Segregation Followed by DNA Fragmentation in Roscovitine-Treated MCF-7 Cells Supports Its Pro-Apoptotic Potential

Kotala V. 1
Horky M. 2
Uldrijan S. 1
Strnad M. 3
Vojtesek B. 1
1 Masaryk Memorial Cancer Institute, Brno, Czech Republic
2 Dept. of Pathological Physiology, Masaryk University, Brno, Czech Republic
3 Lab. of Growth Regulators, University of Palacky, Olomouc, Czech Republic
Publication date: 2001
Electronic publication date: 2001 Oct 18
Volume: 1
First page: 58
Last page: 58
Copyright: Copyright © 2001 V. Kotala et al.
Copyright year: 2001
License: This is an open access article distributed under the Creative Commons Attribution License, which permits unrestricted use, distribution, and reproduction in any medium, provided the original work is properly cited.
License URL: https://creativecommons.org/licenses/by/3.0/

Funding: Grant Agency of Czech Republic 312/99/1550 Ministerstvo Zdravotnictví Ceské Republiky NC6404-3/2000

==============================
Miami *Nature Biotechnology* Short Reports
*TheScientificWorld* (2001) 1 (S3), 124SR
ISSN 1532-2246; DOI 10.1100/tsw.2001.226

### RAPID ONSET OF NUCLEOLAR SEGREGATION FOLLOWED BY DNA FRAGMENTATION IN ROSCOVITINE-TREATED MCF-7 CELLS SUPPORTS ITS PRO-APOPTOTIC POTENTIAL

Kotala, V.[1], Horky, M.[2], Uldrijan, S.[1], Strnad, M.[3], and Vojtesek, B.[1]

[1]Masaryk Memorial Cancer Institute, Brno, Czech Republic; [2]Dept. of Pathological Physiology, Masaryk University, Brno, Czech Republic; [3]Lab. of Growth Regulators, University of Palacky, Olomouc, Czech Republic

**INTRODUCTION.** Nucleolus, previously thought to be solely involved in synthesis of ribosomes, is now reconsidered to be a plurifunctional organelle regulating cellular lifespan and signaling pathways of the programmed cell death (PCD). Rapid proteolysis of nuclear and cytoplasmic proteins, a hallmark of initiation phase of apoptosis, has been shown to involve nucleolar proteins (UBF, RNA polymerase etc.). Roscovitine is a new potent inhibitor of the cyclin-dependent kinases (CDK1, CDK2, CDK5). CDKs as well as p53 protein (tumour suppressor gene, frequently mutated in many human tumours) are involved in crucial pathways regulating cell proliferation and apoptosis. We utilized a unique feature of roscovitine to stimulate cell death in each phase of the cell cycle whereas the DNA damaging drug kills more efficaciously S-phase cells. We focused on the integrity of nucleoli and DNA in roscovitine-treated MCF-7 cells in order to reveal a potential pro-apoptotic effect of roscovitine.

**METHOD.** Human breast carcinoma cell line MCF-7 was cultured in Dulbecco's Modified Eagle Medium (DMEM) supplemented with 10% fetal bovine serum. Cells were treated with 20μM roscovitine. Expression of proteins p53, p21 were characterized using polyacrylamide gel electrophoresis and immunoblotting. For detection of AgNORs, we used the silver staining technique described by Smith et al. (1993). The extent of apoptosis in studied cell line was assessed by TUNEL assay (Boehringer Mannheim, Germany).

**RESULTS.** We studied morphological changes of nucleoli in roscovitine-treated MCF-7 cells and compared these data with TUNEL staining. Untreated cells contained intact nucleoli in comparison with roscovitine-treated cells (6, 12 and 24 hours), where small nucleolar fragments scattered across the whole nucleus were found. Using immunoblotting technique, we also characterized expression of p53 and p21 proteins in MCF-7 cells after 6,12 and 24 hours treatment with roscovitine. This inhibitor effectively induced wild-type p53 protein in MCF-7 cells followed by induction of p21 protein.

**DISCUSSION.** We detected remarkable segregation of silver-stained nucleolar proteins and RNA that preceded TUNEL positivity in roscovitine-treated MCF-7 cells. Our morphological findings as well as the biochemical analysis of p53 and p21 proteins support the view that the pharmacodynamics of potential anti-cancer drug roscovitine may be based on induction of apoptosis.

**ACKNOWLEDGEMENT.** This work was supported by grants: 312/99/1550 Grant Agency of Czech Republic and NC6404-3/2000 Internal Grant Agency of Ministry of Health, Czech Republic.

**REFERENCES.**

1. El-Deiry, W.S., Tokino, T., Velculescu, V.E., Levy, D.B., Parsons, R., Trent, J.M., Lin, D., Mercer, W.E., Kinzler, K.W., and Vogelstein, B. (1993) Cell 75, 817-825
2. Levine, A.J. (1997) Cell 88, 323-331
3. Vesely, J., Havlicek, L., Strnad, M., Blow, J.J., Donella-Deana, A., Pinna, L., Letham, D.S., Kato, J., Detivaud, L., Leclerc, S., and Meijer, L. (1994) Eur. J. Biochem. 224, 771-786
4. Hernandez-Verdun, D. (1991) J. Cell Sci. 99, 465-471
5. Yung, B.Y.M., Bor, A.M.S., and Chan, P.K. (1990) Cancer Res. 50, 5987-5991
